# Factors influencing job performance of nurses in COVID-19 care: a study in Egypt

**DOI:** 10.1186/s12912-024-02479-7

**Published:** 2024-11-19

**Authors:** Ayman Muhammad Kamel Senosy

**Affiliations:** 1 Faculty of Nursing, Ain Shams University, Cairo, Egypt; 2 Faculty of Nursing, El Galala University, Suez, Egypt

**Keywords:** Job Performance, Factors, Nurses’ Care, COVID-19

## Abstract

Coronavirus disease (COVID-19) is an illness that has critical effects on public health and overall circumstances across the world. The performance of nurses represents the vital actions in providing care for COVID-19 patients. The aim of this study was to assess job performance and associated nurses, patients, and environmental-related factors among nurses providing care to COVID-19 patients in Egypt through the following: assessing the nurses’ knowledge regarding care of COVID-19 patients; assessing the nurses’ practice regarding care of COVID-19 patients; assessing different factors that affect different types of nurses' job performance; and assessing the factors associated with the job performance of nurses providing care to COVID-19 patients.

**Design** A descriptive exploratory study was used to meet the aim of the study.

**Setting** The study was conducted at Ain Shams University COVID-19 Hospital.

**Study subjects** A convenient sample of all 52 available nurses working at Ain Shams University COVID-19 Hospital. Data collection tools: The data was collected using a self-administered questionnaire. It consisted of the following: A. Self-administered nurses’ questionnaire: Part I: Demographic characteristics of nurses. Part II: Nurses knowledge assessment. Part III: Factors associated with nurses' job performance. B. Narses’s practice observational checklist. Results: The mean age (mean ± SD) of the study population was 40.59 ± 6.90. As regards their years of experience working as nurses, only 32.7% of them have more than ten years of experience. In relation to the total satisfactory level of knowledge about coronavirus, the study reveals that 55.8% of the nurses have unsatisfactory knowledge regarding coronavirus, There is a highly significant relationship between level of practice and years of experience (*p*-value < 0.001**), while there is a significant relationship between level of practice and educational level and attending the training courses about caring for COVID-19 patients (*P*-value 0.033*, *P*-value 0.025*), respectively.

**Conclusion** The study concluded that most of the studied nurses had unsatisfactory knowledge, and more than half had incompetent practice scores about nursing care practices for patients with COVID-19. There was a positive linear correlation between the nurses' knowledge and practice. The factors affecting their performance in caring for patients with COVID-19, lack of cooperation between nurses and patients, lack of supplies and equipment during care were the most frequently reported factors, as were an unequal nurse-patient ratio, many nursing tasks, and a lack of patients' knowledge.

**Recommendation** The study recommends the importance of promoting the nursing care guidelines as a standard procedure in ICUs, and the nursing care guideline educational program for patients with COVID-19 should be scheduled regularly for nurses. Providing the hospitals with supplies to avoid the shortage's effect on nurses’ performance.

**Supplementary Information:**

The online version contains supplementary material available at 10.1186/s12912-024-02479-7.

## Introduction

Coronavirus disease (COVID-19) is an illness that has a vital effect on public health and overall circumstances across the world. In Wuhan, China, in November 2019, a sickness resembling pneumonia appeared, which was later named by the World Health Organization (WHO) as Coronavirus Disease 2019 or COVID-19. Fear of COVID-19 has been interpreted as a perceived threat to the disease, which has produced vital financial destruction and crushed community mental health in all countries [1].

The COVID-19 pandemic has had a profound effect on different aspects of delivering health care. The SARS-CoV-2 virus has infected more than 350 million people, resulting in more than 5.59 million deaths worldwide [2]. A negative impact across all healthcare sectors has been created due to the COVID-19 pandemic. Nurses faced big challenges at their work areas. They had to adapt to novel current situations, ensure safe delivery of care, and make critical decisions. Also, nurses' managers are responsible for the engagement of their staff nurses who struggle to deal with the new working environment and cope with it, especially staff nurses taking on new roles and tasks that were not done before [3].

They found that most of the components of performance reviews were unsuitable and had been discarded during the coronavirus crisis. So, how execution evaluation tired the current circumstance of COVID-19 becomes a basic issue that ought to be considered within the display time of emergencies [1]. The performance of nurses is very important in providing care for COVID-19 patients. Nurses usually experience a variety of stressful situations. Excessive stress at work influences the mental and physical health of nurses and minimizes their professional efficacy and quality of life. Moreover, high levels of psychological stress can produce job strain and job dissatisfaction [4].

In addition, the nurse is considered one of the leading and vital professions in facing the COVID-19 pandemic. Nurses can reduce complications by taking actions beginning with screening, assessing, emergency, critical care, isolation treatment, monitoring hemodynamics, closely observing the condition of the patient, airway assessment, positioning the patient, and giving education to control critical cases in collaboration with the medical team [5]. The duties fulfilled by the nurse require support from the hospital administration and management to maintain the quality of services at the hospital during the COVID-19 pandemic.

Training programs for nurses include educating and assessing patients, providing care, supervising, monitoring, and coordinating the care process. The concepts of care training mainly include observing and measuring vital signs, inserting, caring for, and removing catheters, changing bandages, and basically giving holistic care according to the needs of the patient. If the nurse does the training, the performance of the nurse is considered good and may be accounted for [6]. There were more studies aimed at finding out the role or influence of engagement motivation and training provided by the hospital as an organization on the nurses' performance during the COVID-19 pandemic era. It is important to provide concise input for human resource management and policymakers in nursing on how to support nurses physically and psychologically to maintain and improve their performances as nurses who are effectively involved during the pandemic [7].

### Significance of the study

Nurses’ performance is highly needed to control and care in the era of the COVID-19 pandemic makes nurses a valuable human resource or employee asset because they can maintain the quality of the hospital when the nurse’s performance is good or damage the hospital's reputation when the nurse’s performance is terrible and can directly impact profits. Since the onset of coronavirus disease in November 2019, many studies have been conducted and published to monitor the effects of disease outbreaks on nurses. During the COVID-19 epidemic, the medical working environment is highly stressful, especially for the nurses. It is important to assess occupational stress, job performance, satisfaction, and intent to leave among nurses caring for COVID-19 patients. [8].

Nurses’ performance is influenced by various aspects, starting from the organizational system in developing potential and opportunities through training programs and attachments to develop and master the field of work to motivational factors in doing their work. [9]. COVID-19 can potentially cause long-term effects on nurses’ performance and job satisfaction, leading to frequent absenteeism and turnover, resulting in psychological distress, job satisfaction, and the intention to leave the organization and the organization and the profession [10].

The performance can serve as a benchmark for enhancing health services to maintain the quality of health services given to sick and healthy patients (Glady et al., 2019). The performance of nurses must be evaluated to maintain quality, determine and plan important career development strategies, and achieve the goals of the organization, especially in the COVID-19 pandemic's era [11]. Nurses’ performance is influenced by many aspects, starting from the organizational system in developing potential and opportunities through programs of training and attachments to master and develop the field of work to motivational factors in doing their work [12].

### Aim of the study

The present study was conducted to fulfill the following aim:

Assess job performance and associated factors among nurses providing care to COVID-19 patients in Egypt through the following:

1. Assessing the nurses' knowledge regarding the care of COVID-19 patients.
2. Assessing the nurses' practice regarding the care of COVID-19 patients.
3. Assessing different factors that affect different types of nurses' job performance
4. Assessing the factors associated with the job performance of nurses providing care to COVID-19 patients

### Research questions

- What was the nurses' knowledge regarding the care of COVID-19 patients?
- What was the nurses' level of practice regarding the care of COVID-19 patients?
- What were the different factors that affected different types of nurses' job performance?
- What were the factors associated with the job performance of nurses providing care to COVID-19 patients?

### Subjects & methods

The present study was conducted to fulfil the following aim:

This study aimed to assess job performance and associated factors among nurses providing care to COVID-19 patients in Egypt through the following:

1. Assessing the nurses' knowledge regarding the care of COVID-19 patients.
2. Assessing the nurses' practice regarding the care of COVID-19 patients.
3. Assessing different factors that affect different types of nurses' job performance
4. Assessing the factors associated with the job performance of nurses providing care to COVID-19 patients

### Research design

A descriptive exploratory study design will be used to achieve the aim of the present study.

#### Setting

The study was conducted in Ain Shams University COVID-19 Hospital, which is affiliated with Ain Shams University Hospitals, Cairo Governorate, Egypt, which is contracted to care for only patients with COVID-19 and is considered one of the biggest hospitals in Egypt.

#### Subjects

A convenient sample of all 52 available nurses working at Ain Shams University COVID-19 Hospital.

**Clinical trial number**: not applicable

Tools for data collection:

A- The data was collected using a self-administered questionnaire. It consisted of the following:

- Part I: Demographic characteristics of nurses:

It was developed by the researcher and has been assessed for reliability and validity based on reviewing relevant literature [13, 14]. Validity and reliability were tested. (Which included age, sex, marital status, level of education, years of experience, and specified departmental questions.).

- Part II: Nurses knowledge assessment.

It was developed by the researcher based on a recent literature review [14, 15]. It was used to assess nurses’ knowledge regarding the care of COVID-19 patients. It was composed of 9 questions, which were distributed as follows: concept of coronavirus, signs, and symptoms The scoring system was adopted, with ratings ranging from 0 (no) to 1 (yes) for each item. Each question's response was either no (0 grade) or yes (1 grade). The total scores of the 9 questions were 9 degrees, which equals 100%. Each question was assigned a score according to the nurses' knowledge responses, which were yes answers scored with 1 and no answers scored with 0. These scores were summed and converted into a percent score. It was classified into two categories:

- satisfactory knowledge if the total score is ≥ 75%.
- Unsatisfactory knowledge if the total score is < 75%

- Part III: Factors associated with nurses’ job performance.

It was developed by the researcher based on a recent literature review [14, 15]. It was used to assess associated factors on nurses’ job performance during providing care for COVID-19 patients. It was divided into three parts: factors related to nurses (10) items; factors related to patients (8) items; and factors related to the environment (7) items. The scoring system is: if the factor present is marked yes and scored (1), and if not, it is marked no and scored (0).

### B – Nurses’ practice observational checklist

It was developed by the researcher based on a recent literature review [4–16]. It was used to assess nurses’ level of practice regarding the care of COVID-19 patients. It included five practical procedures regarding the care of patients with COVID-19. And each procedure had steps.

#### Scoring system

The scoring system for the nurses’ observational checklist was as follows:

One grade for each procedure that was done correctly, zero for procedures that were not done, with a total grade of 5 for the total of (5) procedures.

The total level of nurses’ practice score was categorized as follows:

-  ≥ 70% was considered satisfactory.
-  < 70% was considered unsatisfactory.

#### Operational design

The operational design included the preparatory phase, validity and reliability, a pilot study, ethical considerations, and fieldwork.

#### Preparatory phase

It included a review of the current and more recent relevant national and international literature and theoretical knowledge of various aspects of the study using articles, periodicals, magazines, and books to develop tools for data collection to assess associated factors on nurses’ job performance during providing care for COVID-19 patients. It will include developing tools based on review literature,

The tool was translated from English into Arabic, and back translation was done.

#### Validity and reliability

Validity was ascertained by a group of (5) experts: (3) experts at the faculty of nursing at Ain Shams University, and (2) medical consultants of the Ain Shams University COVID-19 Hospital. Their opinions were elicited regarding the format, layout, consistency, accuracy, and relevance of the tools (Table 1) [17].

**Table 1:** Expert's judgment regarding

Tool Characteristics	Agree	Agree with modifications	disagree
1- Content is related to objectives	100%	0%	0%
2- Content is comprehensive	80%	20%	0%
3- Content is represented	100%	0%	0%
4- Questions are in a logical consequence	80%	20%	10%
5- Content is appropriate	80%	0%	20%
6- Content is accurate	80%	0%	20%
7- Content is clear	60%	40%	0%

#### Reliability

The tools of data collection were tested for its reliability by using Cronbach’s Alpha test in statistical package for social science (SPSS) version 20.

The ethical research considerations in the study included the following:

- The researcher clarified the objectives and aim of the study for the nurses included in the study.
- Nurses' written consents to participate in the study were obtained.
- The names of the studied nurses were not used in the study results.
- The researcher assured maintaining the anonymity and confidentiality of subjects’.
- Nurses were informed that they are allowed to withdraw from the study at any time without any pressure.

#### Field work

The sampling and data collection were started and completed within 9 months. The research lasted around 9 months, from January 2022 to August 2022. Prior to any data collection, the study's purpose was simply described to the nurses who accepted to participate. The study was carried out through this phase.

This phase started at the Ain Shams University COVID-19 hospital by interviewing 52 nurses who care for patients with COVID-19 to explain the aim and nature of the study, as well as getting their approval to participate in the study prior to data collection.

The study tools were filled in and completed by the researcher. The research used clear tools provided by multiple kinds of information from different ways and using certain strategies in framing questions to avoid potential biases in collecting data from the participants.

The nurses’ assessment sheet was used to determine the associated factors on nurses’ job performance during providing care for COVID-19 patients as follows:

Using the nurses’ interview questionnaire to assess nurses' knowledge, which was filled out by the researcher and nurses, it took about 10 min to fill it out for every nurse. Then the observational checklist was used to assess nurses’ performance regarding caring for patients with COVID-19. It took about 20–30 min for every nurse to be fulfilled by the researcher. Then factors affecting nurses’ job performance assessment tool were filled in by the researcher and nurses; it took about 10 min to fill it in for every nurse.

Data collection was done three days a week (Sunday, Monday, and Thursday) at the previously mentioned settings in the morning and afternoon shifts.

#### Administrative design

An official letter was issued from the Nursing Director of the Ain Shams University COVID-19 Hospital at which the study was conducted, explaining the purpose of the study and obtaining their permission to conduct it.

#### Statistical design

Recorded data were analyzed using the statistical package for social sciences, version 23.0 (SPSS Inc., Chicago, Illinois, USA). The quantitative data were presented as mean ± standard deviation. Also, qualitative variables were presented as numbers and percentages.

### The following tests were done

A chi-square (× 2) test of significance was used to compare proportions between qualitative parameters.

Pearson's correlation coefficient (r) test was used to assess the degree of association between two sets of variables.

The confidence interval was set to 95%, and the margin of error accepted was set to 5%. So, the *p*-value was considered significant as follows:

Probability (*P*-value).

- A *P*-value-value was considered significant.
- A *P*-value-value was considered highly significant.
- A *P*-value-value was considered insignificant.

## Results

The present study was conducted to fulfil the aim of the study:

Assess job performance and associated factors among nurses providing care to COVID-19 patients in Egypt.

Table 2 reveals the demographic characteristics of the studied nurses. The present study shows that the mean age (mean ± SD) of the study is 40.59 ± 6.90. Also, 56.3% of patients are in the ward, and 75% of nurses are between 26 and 45 years old. And 73.1% of them are female. As regards the years of experience, only 32.7% of them have more than ten years of experience. In relation to the educational level, only 9.6% of the studied nurses have a bachelor's degree. Also, regarding attending training courses about caring for COVID-19 patients, the study reveals that 75% of the studied nurses do not attend or receive training about caring for COVID-19 patients.

**Table 2:** Sociodemographic characteristics of the studied nurses

Items	Studied Nurses (*N* = 52)	
	N	%
Unit of work (Patients *N* = 71)		
Ward	40	56.3
ICU	31	43.7
Age:		
≤ 25	8	15.4
26- ≥ 45	39	75.0
≥ 46	5	9.6
Mean ± SD	40.59 ± 6.90	
Gender		
Male	14	26.9
Female	38	73.1
Educational Level:		
Diploma	27	51.9
Institute	20	38.5
Bachelor	5	9.6
Marital status:		
Single- Widow- Divorced	15	28.8
Married	37	71.2
Years of experiences:		
> 5 years	16	30.8
5- 10 years	19	36.5
< 10 years	17	32.7
Training courses about caring of COVID 19 Patients		
Yes	13	25.0
No	39	75.0

Table 3 shows the percentage distribution of nurses’ knowledge about coronavirus. The study indicates that only 53.8% of them know the concept of coronavirus, 46.2% of the studied nurses have knowledge about the common signs and symptoms, 40.4% of them know the prompt treatment for all disease stages, and 44.2% of them have knowledge about the treatment of infected patients with chronic diseases. In relation to the total satisfactory level of knowledge about coronavirus, the study reveals that 55.8% of the respondents have unsatisfactory knowledge regarding coronavirus.

**Table 3:** Percentage distribution of nurses’ knowledge about coronavirus

Item	Studied Nurses (*N* = 52)			
	Satisfactory (Yes)		Unsatisfactory (No)	
	N	%	N	%
Concept of coronavirus	28	53.8	24	46.2
Symptoms and signs	24	46.2	28	53.8
Causes of infection	25	48.1	27	51.9
Complications	26	50.0	26	50.0
Types of virus mutation	18	34.6	34	65.4
Preventive measures	27	51.9	25	48.1
Prompt treatment for all disease stages	21	40.4	31	59.6
Treatment of infected patients with chronic diseases	23	44.2	29	55.8
Types of vaccines	18	34.6	34	65.4
Total	23	44.2	29	55.8

**Fig. 1:**
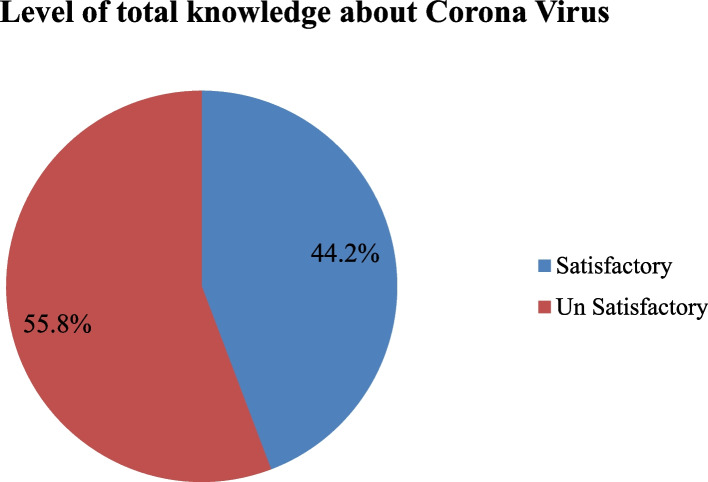
Percentage distribution of nurses’ knowledge about coronavirus

Regarding the distribution of nurses’ knowledge about coronavirus, Fig. 1 reveals that 44.2% have a satisfactory level of knowledge, while 55.8% of the studied nurses have an unsatisfactory level of knowledge.

Table 4 presents the distribution of the studied nurses' related factors. The current study represents that 63.5% and 65.4%, respectively, of the studied nurses reported that factors related to nurses during providing care are the unbalanced nurse-patient ‘ratio and a lack of nurses' knowledge and experiences. While 73.1% of nurses related factors are work overload and 78.8% are due to increasing nurses’' duties, Also, only 53.8% of the nurses’ have psychological pressures, and 44.2% have family ‘issues. While 30.8% of them suffer from lack of self-confidence.

**Table 4:** Distribution of studied nurses about nurse’ related factors

Nurse’ related factors	Studied Nurses (*N* = 52)			
	( Yes)		(No)	
	N	%	N	%
Un equal nurse patient’s ratio	33	63.5	19	36.5
Lack of nurse’s knowledge and experiences	34	65.4	18	34.6
Lack of supervision	20	38.5	32	61.5
Lack of coordination between staff	18	34.6	34	65.4
Work overload	38	73.1	14	26.9
Nurses’ having psychological pressure	28	53.8	24	46.2
Differences of educational level	29	55.8	23	44.2
Increase nurses’ duties	41	78.8	11	21.2
Nurses’ family issues	23	44.2	29	55.8
Lack of nurses’ self confidence	16	30.8	36	69.2

Table 5 reveals the distribution of the studied nurse’s knowledge about the patients’ related factors. There are (76.9% and 75%, respectively) factors related to patients due to the lack of patients’ knowledge and the patients with psychological distress. Also, 71.2% of the study is related to the lack of patients’ awareness about preventive measures, and only 42.3% of the study shows that the patients do not follow the nurses’ instructions.

**Table 5:** Distribution of studied nurses about Factors related to patients

Patients’ related factors	Studied Nurses (*N* = 52)			
	( Yes)		( No)	
	N	%	N	%
Lack of patients’ knowledge	40	76.9	12	23.1
Patients with other chronic diseases	32	61.5	20	38.5
Patients with psychological distress	39	75.0	13	25.0
Patients in isolation state	29	55.8	23	44.2
Lack of communication between nurses and patients	30	57.7	22	42.3
Patients have wrong concepts and beliefs	31	59.6	21	40.4
Lack of patients’ awareness about preventive measures	37	71.2	15	28.8
Patients not follow nurses’ instructions	22	42.3	30	57.7

Table 6 displays the distribution of the studied nurses' environmental'-related factors. The nurses reported that factors that affect them are 59.6% lack of supplies and (67.3% and 61.5%), respectively, lack of treatment and the overcrowding of rooms with patients. And 40.4% and 34.6% were as result of lack of physician and lack of clean environment. Also, only 53.8% is due to the restricted policies of the hospital, which interrupt work. While 36.5% was due to the barriers of communication with physicians.

**Table 6:** Distribution of studied nurses about environmental Factors

Environmental related factors	Studied Nurses (*N* = 52)			
	(Yes)		(No)	
	N	%	N	%
Lack of supplies	31	59.6	21	40.4
Lack of treatment	35	67.3	17	32.7
Overcrowded room with patients	32	61.5	20	38.5
Lack of physicians	21	40.4	31	59.6
Lack of clean environment	18	34.6	34	65.4
Restricted policies of hospital causing interrupting work	28	53.8	24	46.2
Barriers of communication with physicians	19	36.5	33	63.5

Table 7 shows the percentage distribution of nurses’ practices regarding during care patients with coronavirus disease. 59.6% of the studied nurses have skills in performing hand washing, and 34.6% and 36.5%, respectively, can do respiratory hygiene, cough etiquette, and safe injection practices. In total practice, only 48.1% have a satisfactory level of practice.

**Table 7:** Percentage distribution of nurses’ practices regarding during care patients with coronavirus disease

Practices (Skills)	Studied Nurses (*N* = 52)			
	Satisfactory (Done)		Un satisfactory (Not done)	
	N	%	N	%
Hand Washing	31	59.6	21	40.4
Using personal protective equipment	30	57.7	22	42.3
Respiratory hygiene / cough etiquette	18	34.6	34	65.4
Safe injection practices	19	36.5	33	63.5
Sterile instruments and devices	25	48.1	27	51.9
Total	25	48.1	27	51.9

**Fig. 2:**
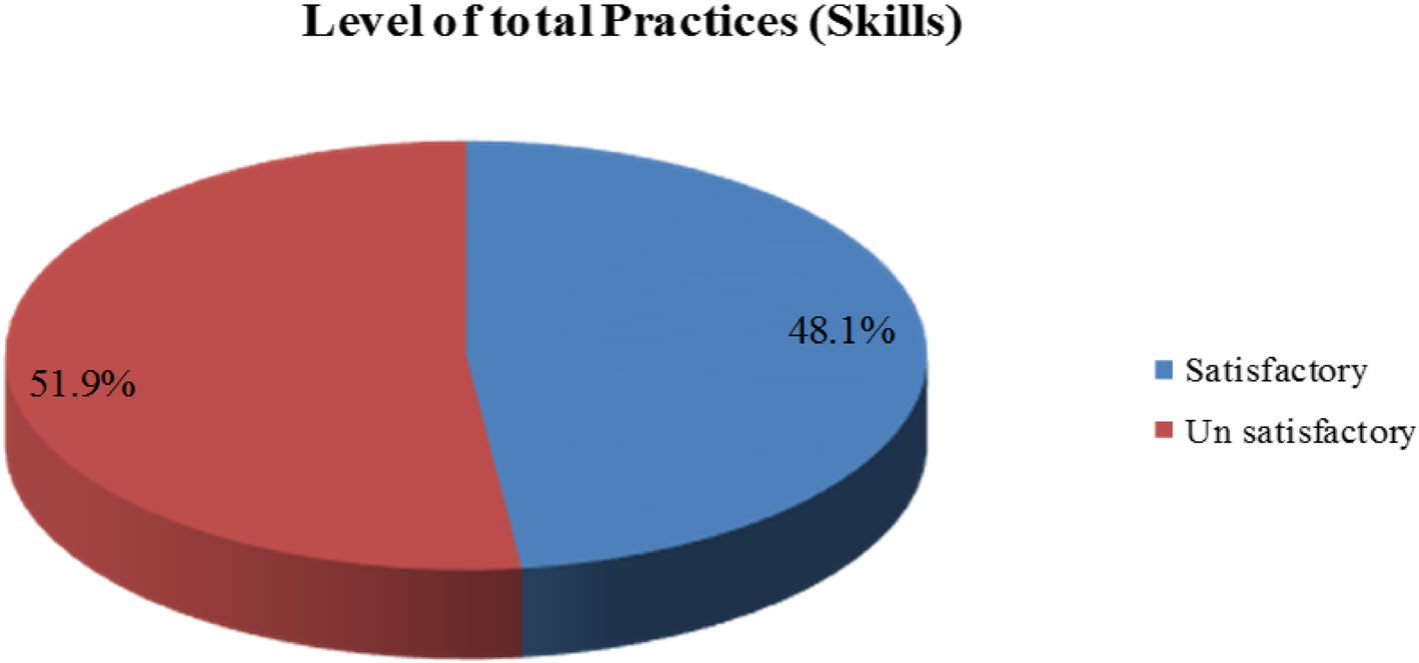
Percentage distribution of nurses’ practices regarding during care patients with coronavirus disease

Figure 2 of nurses’ practices regarding patients with coronavirus disease reveals that 48.1% of nurses have a satisfactory level, while 51.9% have an unsatisfactory level.

Table 8 shows the relation between the level of practice (performance) of coronavirus among the studied nurses according to their socio-demographic data characteristics. There is a highly significant relationship between level of practice and years of experience (*p*-value < 0.001**), while there is a significant relationship between level of practice and educational level and attending the training courses about caring for COVID-19 patients (*P*-value 0.033*, *P*-value 0.025*), respectively.

**Table 8:** Relation between level of practice (performance) of coronavirus of the studied nurses according to their socio-demographic data characteristics

Socio-demographic data characteristics	Level of performance				Chi-square test	
	Satisfactory (*n* = 24)		Unsatisfactory (*n* = 28)			
	No	%	No	%	× 2	*p*-value
Age:						
≤ 25 (8)	4	16.7	4	14.3	0.537	0.765
26- ≥ 45 ( 39)	17	70.8	22	78.6		
≤ 46 (5)	3	12.5	2	7.1		
Mean ± SD						
Educational Level:						
Diploma (27)	8	33.3	19	67.9	6.814	0.033*
Institute (20)	12	50.0	8	28.6		
Bachelor (5)	4	16.7	1	3.6		
Years of experiences:						
> 5 years (16)	4	16.7	12	42.9	18.003	< 0.001**
5- 10 years (19)	5	20.8	14	50.0		
< 10 years (17)	15	62.5	2	7.1		
Training courses about caring of COVID 19 Patients						
Yes ( 13)	10	41.7	3	10.7	5.056	0.025*
No (39)	14	58.3	25	89.3		

Using: Chi-square test & Fisher’s Exact test

*p*-value > 0.05 NS; *p-value < 0.05 S; **p-value < 0.001 HS

Table 9 represents the correlation between the total score of performance of the studied nurse according to their total score of factors. There are highly statistically significant differences between the total score of performance (practice) with the nurse's related factors and environmental factors (*p*-value < 0.001** for both).

**Table 9:** Correlation between total score of performance of the studied nurse according to their total score of factors

	Total score Performance (Practice)		
	N	*r*-value	*p*-value
Nurse’ related factors	52	0.627	< 0.001**
Factors related to patients	52	0.481	0.005*
Environmental Factors	52	0.543	< 0.001**

r-Pearson Correlation Coefficient

^**^Highly statistically significant differences (*p* < 0.001)

^*^A statistically significant difference (*p* < 0.05)

## Discussion

Regarding the demographic characteristics of the studied nurses, the present study showed that the mean age (mean ± SD) of the study was 40.59 ± 6.90. Also, more than half of the patients were in the ward, and three-quarters of the nurses their ages were between twenty-six years and less than forty-five years. And three-quarters of them were female. As regards the years of experience, only about one-third of them had more than ten years of experience. Also, regarding attending training courses about caring for COVID-19 patients, the study revealed that three-quarters of the studied nurses did not attend or receive training about caring for COVID-19 patients.

These findings are consistent with those of [18] in their study entitled Occupational stress, job satisfaction, and intent to leave: nurses working on the first lines during the COVID-19 pandemic in Zagazig City, Egypt, who found that the majority of the participants in both groups were females, and the mean age of the ZFH Group was 24.22 ± 5.16, which was significantly lower than 26.09 ± 4.53 for the Zagazig General Hospital Group.

Regarding the nurses' knowledge about coronavirus, the study revealed that less than two-thirds of them know the concept of coronavirus, less than half of the studied nurses have knowledge about the common signs and symptoms, only two-fifths of them know the prompt treatment for all disease stages, and less than half of them have knowledge about the treatment of infected patients with chronic diseases. In relation to the total satisfactory level of knowledge about coronavirus, the study revealed that more than half of the participants had an unsatisfactory level of knowledge regarding coronavirus.

These findings are consistent with [19] study entitled Nurses' knowledge, concerns, perceived impact, and preparedness towards the COVID-19 pandemic: A cross-sectional survey. Most of the studied nurses knew the nature of the virus and its symptoms and signs, such as fever, cough, and dyspnea, and most of them knew that people with chronic diseases and the elderly are more likely to be infected.

Add to that, these findings are related to [20], in their study entitled “Factors Affecting the Caring Performance of Newly Graduated Nurses' Working in Critical Care Units," which confirms the importance of various recommendations and implications for nursing practice, education, administration, and research. For practice, paying more attention to work-related stress by nursing management to reduce it might influence nurses' ability to provide higher-quality nursing care, respond to patients' needs in critical care units, and decrease their intent to quit their jobs and their burnout. [21]. While for education, there is a need to emphasize, enhance, and integrate the concept of caring as the essence of the nursing profession in nursing curricula. In-service education programs and continuing education programs may adopt specific training programs to promote the caring performance of all nurses working in critical care Also, nursing researchers might require employing qualitative research designs to provide in-depth information from the perspectives of the newly graduated nurses employed in critical care units [22].

Regarding the distribution of the studied nurses' related factors, the current study indicates that nearly two-thirds of the studied nurses reported that factors related to nurses during providing care were the unbalanced nurse-patient ‘ratio and a lack of nurses' knowledge and experiences. While nearly three-quarters of nurses related factors were work overload and increasing nurses’' duties, Also, more than half of them had psychological pressure, and more than one-third of them had family ‘issues.

These findings are consistent with the [23] study entitled A closer look at the high burden of psychiatric disorders among healthcare workers in Egypt during the COVID-19 pandemic. Who focused on the fact that the COVID-19 pandemic has had a negative effect on the health care workers' psychological well-being in Egypt? They need psychological support, and preventive measures should be implemented to prevent the further progress of psychiatric illness among health care workers.

These findings are also consistent with the confirmation that lowering the nurse-to-patient ratio is associated with better outcomes for patients and improved safety in critical care units [24]. Also, a higher ratio poses a challenge for nurses to provide high-quality care for more than one patient and would elevate nurses' turnover intentions [25]. Add to that, about two-thirds of the participants worked a rotating shift, indicating an unstable work schedule among most of the nurses, which might cause high levels of work-related stress [26].

Regarding the level of work-related stress, the current study found that the participants had a high level of work-related stress in all aspects of MHPSS, which is consistent with previous literature examining work-related stress among nurses working in CCUs [27, 28].

In relation to the distribution of the studied nurses' related factors, the study showed that about three-quarters of factors related to patients were due to a lack of patients’ knowledge and patients with psychological distress. Also, more than two-thirds of the study was related to the lack of patients’ awareness about preventive measures, and less than half of the study found that the patients did not follow the nurses’ instructions.

These findings are in contrast with those of [29], in their study entitled Fear of the COVID-19 Scale: Psychometric Properties, Reliability, and Validity in the Egyptian Population. Who stated that healthcare workers have more contact with patients frequently, they can categorize mental and psychological health issues, especially as an effect of COVID-19, and provide treatment and care based on that.

As regards the distribution of the studied nurses' environmental-related actors, The nurses reported that factors that affected them were nearly two-thirds due to the lack of supplies, and more than half were the result of the lack of treatment and the overcrowding of rooms with patients. Also, nearly three-fifths were due to the restricted policies of the hospital, which interrupted work.

These findings are consistent with those of [30], in their study entitled Predictive factors affecting stress among nurses providing care at COVID-19 isolation hospitals in Egypt. Who reported that the availability of PPE, educational level, training for COVID-19, and attention to hospital policies and administration were negative predictor factors for nurses’ stress?

In fact, work-related stress was not only associated with decreased caring performance but also with various negative consequences for psychological and physical health status [31]. Unmanaged work-related stress can reduce nurses' productivity and ability to provide patient care, negatively affect the overall organizational structure and its internal stability and be detrimental to the quality of patient care [32]. Add to that, high levels of work-related stress may lead to an elevated turnover rate and illness [25–33]. Therefore, controlling work-related stress could enhance nursing performance and the overall quality of nursing care.

Regarding the percentage distribution of nurses’ practices regarding during care patients with coronavirus disease, only more than half of the studied nurses had skills of performing hand washing, and nearly one-third had skills of doing respiratory hygiene, cough etiquette, and safe injection practices. In total, less than half of the studied nurses had a satisfactory level of practice.

These findings are related to a [34] study entitled “All of this was awful." exploring the experience of nurses caring for patients with COVID-19 in the United States. Who found that the nurses working with patients with COVID-19 felt unprepared, helpless, and overwhelmed? The patient conditions described appeared to have an insatiable need for nurse care, which impacted the well-being of nurses. And focusing on training nurses and improving their skills regarding the care of patients with COVID-19 is important, especially in terms of using personal protective equipment and following infection control measures.

Studies have also shown that organizational factors, nurses’ cooperation with other professions, interpersonal cooperation, and relationships with leaders or supervision influenced their performance. Good relationships and regular supervisory supervision with various parties in the work area will enhance nurses’ ability and motivation to do their duties better. Despite other influencing factors such as salary in accordance with regional minimum wages, motivation, and non-financial rewards, this was in relation to the [35] study, in which non-financial and financial rewards had a positive impact on the performance of health workers. Non-financial rewards can be used to promote candidates for specialized training.

Regarding the relation between level of practice (performance) of coronavirus of the studied nurses according to their socio-demographic data characteristics, the study indicated that there was highly significant relation between level of practice and years of experiences with (*p*-value < 0.001**), while there was significant relation between level of practice and educational level and attending the training courses about caring for COVID-19 patients (*P*-value 0.033*, *P*-value 0.025*) respectively.

These findings are inconsistent with the [36] study entitled “Relation between Nurses' Performance and Their Demographic Data.” The results showed no significant relation between their performance and (age, gender, training course, shift time, source of education).

As regards the correlation between the total score of performance of the studied nurse according to their total score of factors, the study revealed that there were highly statistically significant differences between the total score of performance (practice) with the nurse's related factors and environmental factors (*p*-value < 0.001** for both).

These are consistent with the [37] study, "Associations Among Nursing Work Environment and Health-Promoting Behaviors of Nurses and Nursing Performance Quality: A multilevel modeling Approach,” which indicated that there were significantly associated with more healthy eating among nurses, which is a positive collegial physician-nurse relationship in units. [38]. Nurses with experience person–job match in the value, community, and control areas of work life with self-characters can be more likely to embed in their job. Nurses working in units with sufficient resources and staffing and who had health responsibility and a higher level of spiritual growth were more likely to perceive their nursing practice and performance quality as being higher.

### Limitations of study

Shortage of time: the length of hospital stay during interviewing nurses was very short, in which sometimes the nurses were very busy caring for patients, and they were also wearing PPE that always made them unable to talk well in the shift. Also, a major problem is the heavy workload of hospital nurses, which overloads nurses by caring for more patients in the same shift. The sample size was small 52 participants, but it was convenience that involved all available nurses in the hospital.

## Conclusion

Based on the findings of the present study, it can be concluded that:

The study concluded that most of the studied nurses had unsatisfactory knowledge, and more than half had incompetent practice scores about nursing care practices for patients with COVID-19. There was a positive linear correlation between the nurses' knowledge and practice. The factors affecting their performance in caring for patients with COVID-19, lack of cooperation between nurses and patients, lack of supplies and equipment during care were the most frequently reported factors, as were an unequal nurse-patient ratio, many nursing tasks, and a lack of patients' knowledge.

### Recommendations

Based on the results of the current research, the following suggestions for future research and practice are proposed:

1. Promoting the nursing care guidelines as a standard procedure in ICUs.
2. A nursing care guideline educational program for patients with COVID-19 should be scheduled regularly for nurses.
3. Providing the hospitals with supplies to avoid the shortage's effect on nurses’ performance.
4. Continuous observation of the nurses ‘associated factors and sharing the nurse's role in solving the issues.
5. Strengthening efforts to address the safety of the health and care workforce, including by ensuring priority access to pandemic-related products during pandemics, their means of transport and equipment, as well as hospitals and other medical facilities, while carrying out pandemic prevention and response.

## Supplementary Information

Supplementary Material 1.

## Data Availability

No datasets were generated or analysed during the current study.
