# Supplementary material for: Factors influencing job performance of nurses in COVID-19 care: a study in Egypt
Source: BMC Nurs. 2024 Nov 19;23:846. doi: 10.1186/s12912-024-02479-7 (PMC11577893; doi:10.1186/s12912-024-02479-7)
Supplement: Supplementary file 1 — Supplementary Material 1. [file 12912_2024_2479_MOESM1_ESM.pdf]

Questionnaire to assess nurses' knowledge at Ain Shams University COVID-19 Hospital

Appendix (A)

Part I: Sociodemographic data:

- **Code number/…..**
- **Unit and department**/………
- **Gender**

  a. Male ( ) b. Female ( ).
- **Age**

  a. Less than 25 years ( ) c. from 40 years and more. ( )

  b. from 25 to less than 40 years ( )
- **Marital status**

  a. Married ( ) b. Single- Widow- Divorced ( )
- **Educational Level**

  a. Diploma ( ) b. Institute nursing ( ) c. Bachelor of nursing science ( )
- **Years of experiences:**

  a. Less than 5 years. ( ) c. From 5 to 10 years ( )

  b. More than 10 years. ( )
- **Training courses about caring of COVID 19 Patients**

  a. Yes ( ) b. No ( ).

Part II: Nurses knowledge regarding COVID-19.

Question No.	Do you have knowledge regarding the following.	Yes	No
1	Concept of corona virus		
2	Symptoms and signs		
3	Causes of infection		
4	Complications		
5	Types of virus mutation		
6	Preventive measures		
7	Prompt treatment for all disease stages		
8	Treatment of infected patients with chronic diseases		
9	Types of vaccines		

Part III: Factors associated with nurses' job performance.

- **Nurse` related factors**

No.	**Nurse` related factors**	Yes	No
1.	Un equal nurse patient's ratio		
2.	Lack of nurse's knowledge and experiences		
3.	Lack of supervision		
4.	Lack of coordination between staff		
5.	Work overload		
6.	Nurses` having psychological pressure		
7.	Differences of educational level		
8.	Increase nurses` duties		
9.	Nurses` family issues		
10.	Lack of nurses` self confidence		

- **Patients` related factors**

No.	**Patients` related factors**	Yes	No
1.	Lack of patients' knowledge		
2.	Patients with other chronic diseases		
3.	Patients with psychological distress		
4.	Patients in isolation state		
5.	Lack of communication between nurses and patients		
6.	Patients have wrong concepts and beliefs		
7.	Lack of patients' awareness about preventive measures		
8.	Patients not follow nurses` instructions		

- **Environmental related factors**

**No**	**Environmental related factors**	**Yes**	**No**
1.	Lack of supplies		
2.	Lack of treatment		
3.	Overcrowded room with patients		
4.	Lack of physicians		
5.	Lack of clean environment		
6.	Restricted policies of hospital causing interrupting work		
7.	Barriers of communication with physicians		

Appendix (B)

Nurses' practice observational checklist

No	Procedure	Done	Not done
1	Hand Washing		
2	Using personal protective equipment		
3	Respiratory hygiene / cough etiquette.		
4	Safe injection practices		
5	Sterile instruments and devices.		
